# Satisfaction with job and family life, and association with smoking and alcohol drinking behaviors among young men in Malawi: analysis from a multiple indicator survey

**DOI:** 10.1186/s13104-019-4096-4

**Published:** 2019-01-24

**Authors:** Sanni Yaya, Amos Buh, Ghose Bishwajit

**Affiliations:** 1 School of International Development and Global Studies, Faculty of Social Sciences, University of Ottawa, 120, University Private, Ottawa, ON K1N 6N5 Canada; 2 Interdisciplinary School of Health Sciences, Faculty of Health Sciences, University of Ottawa, 25 University Private, Ottawa, ON K1N 7K4 Canada

**Keywords:** Satisfaction, Job, Overall family life, Alcohol drinking, Smoking, Global health, Malawi

## Abstract

**Objective:**

The objective of the present study was to investigate if satisfaction with job and family life has any connection with smoking and alcohol drinking behavior among young men in Malawi.

**Results:**

Results of multivariable logistic regression analysis indicate that compared to men who were unemployed, those who were dissatisfied were 0.90 times less likely to be non-smokers [OR = 0.90; 95% CI = 0.36–2.24], 0.83 times [OR = 0.83; 95% CI = 0.63–1.08] as likely to try drinking alcohol. Among those who reported being satisfied with job, the odds of trying alcohol was relatively more [OR = 0.77; 95% CI = 0.63–0.93], however the odds of cigarette smoking were less [OR = 1.05; 95% CI = 0.48–2.31] relative to those who were unemployed. Results also showed that not being satisfied with overall life increased the odds of smoking and alcohol drinking [OR = 0.60; 95% CI = 0.24–1.46] and [OR = 0.95; 95% CI = 0.72–1.24] respectively compared to those who were satisfied with overall life.

## Introduction

Smoking and alcohol intake are growing public health concerns. The World Health Organization (WHO) estimates that there are more than 1 billion users of tobacco worldwide, more than 80% of whom are males [1]. In Africa in particular, the gap between male and female smokers is narrowing with more women smoking than before [2]. However, there are gender variations in the prevalence of smoking in different countries; in Malawi, a study has reported that more males (25.9%) compared to females (2.9%) are smokers [3]. In fact, Malawi is one of the sub-Saharan African countries with a higher prevalence of smoking [2, 3].

Concerning alcohol intake, WHO estimates almost 2 billion consumers, 76 million of whom are already experiencing alcohol use disorders [4]. The rate of alcohol consumption in Africa is predicted to be on the increase owing to the emergence of new consumers like young people and women [4]. It has also been noted that the increase in alcohol consumption is high in underdeveloped countries and such increases are likely to hinder further development [5]. Malawi, a developing country in Africa, has little or no available information about alcohol consumption among its population.

Smoking and alcohol intake both have multiple causes. For tobacco use, some documented factors can be grouped into social and physical environmental factors such as religious activity, educational attainment, socioeconomic status, peer influences and cognitive decision-making factors about tobacco use and its consequences [6]. A study has also found parental smoking and the associated nicotine dependence as a risk factor for adolescent smoking [7]. Nevertheless, other studies have reported personality factors, cognitive factors, coping resources, family influences, media influences, tobacco availability, occupational stress, peer pressure and domestic stress as factors underlying smoking habits [8–10].

For alcohol intake, a study has reported that the presence of high antisocial behavior, high impulsivity and high externality are factors related to alcohol dependence specifically for women [11]. Yet, another study has highlighted genetic risk factors, biological markers, childhood behaviors and psychiatric disorders to be associated to alcohol intake [12]. Also, some studies found that students who dropped out of high school were more than 6 times as likely to resort to alcohol use/abuse in adulthood. The studies thus concluded that education as well as race were associated with higher alcohol consumption behaviors [13, 14].

Besides education, wealth index has been often cited as a factor related to smoking and alcohol consumption. In one study, an increased risk of drinking in wealthier individuals was found with a rather lower risk of smoking [15]. In another study, the rise in wealth inequity was independently associated with alcohol drinking problems only [16]. Nonetheless, not only is there uncertainty about factors driving smoking and drinking, literature on this topic is also limited especially in a developing country like Malawi with likely fewer and less paid jobs but larger families. It was thus necessary to investigate if satisfaction with job and family life has any connection with smoking and drinking behaviors among young men in Malawi.

## Main text

### Materials and methods

The study used data from the latest (5th round of the) Multiple Indicator Cluster Survey (MICS) that was conducted in Malawi from 2013 to 14. The MICS program was developed by UNICEF, it is a global initiative, aimed at providing internationally comparable data on various health indicators and it is operational in more than 100 countries. The outcomes of the surveys have been instrumental in monitoring progress towards the millennium development goals (MDGs) and developing health policies and programs to meet internationally agreed commitments by participating nations.

MICSs employ a multistage cluster-sampling strategy to select country representative samples. For the 5th MICS conducted in Malawi, the sample was selected from the Northern, Central and Southern Regions and included both urban and rural areas across 27 districts of the country (excluding Likoma). An English questionnaire that was pretested and then translated into Chichewa and Tumbuka was used for data collection. The Malawi 2013–2014 MICS successfully interviewed 6842 men aged between 15 and 49 years. Further details regarding the survey are published in reports elsewhere [1, 2].

### Variables

Study variables were characteristics of participants from whom data was collected. Main outcome variables were self-reported past or current smoking/alcohol drinking, or both smoking (Yes, No) and alcohol drinking (Yes, No). Explanatory variables were satisfaction with job and life overall (Very satisfied, somewhat satisfied, neither satisfied nor unsatisfied, somewhat unsatisfied, very unsatisfied), Age (15–19, 20–24, 25–29, 30–34, 35–39, 40–44, 45–49); Region (Northern, Central, Southern); Religion (Christian, Islam, Other); Education (None, Primary, Secondary, Higher); Wealth index (Poorest, Second, Middle, Fourth, Richest).

### Data analysis

Data were analyzed with SPSS version 24. Sample characteristics were presented with descriptive statistics such as frequencies and percentages. The proportion of sample population with smoking and drinking habits were calculated using cross-tabulation for all the explanatory variables. Chi squared bivariate tests were used to measure the significance of the associations. Following that, binary logistic regression analyses were run to measure the multivariate association between smoking and drinking with self-reported satisfaction with job and life. The results were reported as odds ratios and 95% CI. P-value were regarded as significant at 5% for all analyses.

### Results

#### Characteristics of study participants

The characteristics of study participants are presented in Table 1. More than half (58.6%) of the participants were aged 15–19 years and 48.2% of them were residing in the southern regions of the country. Majority of the participants (81.1%) were Christians, most of whom (62.4%) had attained only the primary level of education. Seven hundred and thirty-nine (26.2%) of the participants were from households with the richest wealth quintile and 44.6% of them were satisfied with their jobs while 88.5% of them were satisfied with their overall life. About half (50.6%) of the participants were not currently smoking tobacco while 70.8% of them have never drank alcohol.

**Table 1:** Sample characteristics (n = 2824), Malawi MICS 2013–2014

Variables	N	%
*Age*		
15–19	1654	58.6
20–24	1170	41.4
*Region*		
Northern	363	12.9
Central	1100	38.9
Southern	1361	48.2
*Religion*		
Christian	2291	81.1
Islam	418	14.8
Other	115	4.1
*Education*		
None	69	2.4
Primary	1761	62.4
Secondary	927	32.8
Higher	66	2.3
*Wealth index quintile*		
Poorest	472	16.7
Second	511	18.1
Middle	512	18.1
Fourth	591	20.9
Richest	739	26.2
*Satisfaction with job*		
Unemployed	1176	41.2
Unsatisfied	407	14.3
Satisfied	1273	44.6
*Satisfaction with overall life*		
Unsatisfied	327	11.5
Satisfied	2524	88.5
*Currently smoking cigarettes*		
Yes	774	49.4
No	794	50.6
*Ever drunk alcohol*		
Yes	825	29.2
No	1999	70.8

*N* frequency, *%* frequency in percentage

#### Prevalence and correlates of smoking and alcohol drinking among young men in Malawi

Prevalence of smoking and drinking across participants’ characteristics are presented in Table 2. Smoking and drinking was more prevalent among participants who ended their education at primary school level. While participants with the poorest household wealth were more likely to smoke, those with the richest household wealth were more likely to drink. Results of multivariable logistic regression analysis indicate that compared to men who were unemployed, those who were dissatisfied were 0.90 times less likely to be non-smokers [OR = 0.90; 95% CI = 0.36–2.24], 0.83 times [OR = 0.83; 95% CI = 0.63–1.08] as likely to try drinking alcohol. Among those who reported being satisfied with job, the odds of drinking alcohol were significantly higher [OR = 0.77; 95% CI = 0.63–0.93]. Results also showed that not being satisfied with overall life increased the odds of smoking and alcohol drinking [OR = 0.60; 95% CI = 0.24–1.46] and [OR = 0.95; 95% CI = 0.72–1.24] respectively compared to those who were satisfied with overall life (Table 3). However, these findings were not statistically significant.

**Table 2:** Prevalence of past and or currently smoking and alcohol drinking across participants’ characteristics in Malawi (n = 2824)

	Smoking	p	Drinking alcohol	p
*Education*		< 0.0001		< 0.0001
None	5.00		2.40	
Primary	32.60		25.60	
Secondary	11.20		15.20	
Higher	0.50		2.30	
*Wealth index*		< 0.0001		0.041
Poorest	13.50		7.10	
Second	11.60		8.20	
Middle	9.80		8.80	
Fourth	8.20		9.50	
Richest	6.20		11.90	
*Satisfaction with job*		0.651		< 0.0001
Unemployed	6.00		9.40	
Dissatisfied	6.30		4.30	
Satisfied	15.90		14.10	
*Satisfaction with overall life*		0.253		0.585
Dissatisfied	4.40		3.30	
Satisfied	23.50		24.40	

*P* P-value

**Table 3:** Association between satisfactions with job and family life and ever trying smoking, drinking, or trying both among men in Malawi (multivariate regression analysis)

	Smoking	Drinking alcohol
	aOR (95% CI)	aOR (95% CI)
***Unemployed***		
*Satisfaction with job*		
Dissatisfied	0.90 (0.36–2.24)	0.83 (0.63–1.08)
Satisfied	1.05 (0.48–2.31)	0.77 (0.63–0.93)
*Satisfaction with overall life*		
Satisfied		
Dissatisfied	0.60 (0.24–1.46)	0.95 (0.72–1.24)

*aOR* adjusted odds ratio

This study examined the connection between satisfaction with job and family life and smoking and drinking among men in Malawi. Results show that when compared with those without a job, young men who had a job and were satisfied were relatively more likely not to resort to cigarette smoking. However, those who had a job but were dissatisfied were more likely to resort to smoking than those who were without a job. This finding is consistent with those of previous studies which found that the odds of smoking among those who were unemployed were greater compared to higher managers and professionals [17, 18]. Although the precise mechanism by which unemployment is related to smoking has not been well explored in studies, the possible role of psychosocial factors underlying this relationship such as one’s inability to control emotions have been hinted [17].

Regarding the association of satisfaction/dissatisfaction with job and smoking, this study revealed that those who were dissatisfied with their job were more likely to smoke than those who were unemployed. This is also consistent with results of previous studies demonstrating that the impact of working conditions/job dissatisfaction is related with smoking [19, 20]. Our study also revealed that dissatisfaction with overall family life was associated with higher smoking prevalence. This is also consistent with results of similar [20–22]. It is perceived that cigarette smoking relieves the stress arising from dissatisfaction with family life [21, 22].

Our study observed no increase in drinking behavior related to unemployment but only changes in the drinking patterns. This is similar to observations from other studies arguing that unemployment does not lead to alcohol consumption [23, 24]. Also, we found no significant relationship between drinking and overall satisfaction with life. However, it has be suspected that both dissatisfaction with life and unemployment are associated with low socio-economic status which might lead to actual reduction in drinking habit due in part to the economic situation [25–27].

Education and wealth status were consistently associated with both smoking and alcohol consumption. More educated participants were less likely to resort to both smoking and alcohol consumption. Previous studies have reported similar findings, with low education being noted as an independent risk factor for smoking and alcohol intake [28–30].

With regards to wealth index and association with smoking and drinking behavior, non-linear relationships were observed. While the poorest are more likely to resort to smoking and alcohol consumption compared to the poor and middle-income individuals, the relationship changes when wealth index quintile improves; the rich and richest are more likely than middle income and poor household individuals to resort to smoking and drinking. Similar studies have reported these same findings [31, 32].

## Limitations

Our findings might be limited considering the secondary nature of the data we used which allowed us little control over the variables to include in our analysis, and the fact that we assessed only the association of satisfaction with job and family life, with smoking and drinking while omitting variables such as ethnicity, marital status, beliefs, values and attitudes and men’s age which might influence smoking and drinking behaviors. However, we worked within the confines of the objectives and other correlates of smoking and drinking could be explored in another study.

## Abbreviations

aOR: adjusted odds ratio
CI: confidence interval
OR: odds ratio
MDG: millennium development goal
MICS: multiple indicator cluster survey
N: frequency
OR: odds ratio
WHO: World Health Organization
